# Relationship between static foot posture and foot mobility

**DOI:** 10.1186/1757-1146-4-4

**Published:** 2011-01-18

**Authors:** Mark W Cornwall, Thomas G McPoil

**Affiliations:** 1 The Laboratory for Foot and Ankle Research, Department of Physical Therapy and Athletic Training, Northern Arizona University, Flagstaff, AZ 86011, USA; 2 School of Physical Therapy, Regis University, Denver, CO 80221, USA

## Abstract

**Background:**

It is not uncommon for a person's foot posture and/or mobility to be assessed during a clinical examination. The exact relationship, however, between static posture and mobility is not known.

**Objective:**

The purpose of this study was to determine the degree of association between static foot posture and mobility.

**Method:**

The static foot posture and foot mobility of 203 healthy individuals was assessed and then analyzed to determine if low arched or "pronated" feet are more mobile than high arched or "supinated" feet.

**Results:**

The study demonstrated that those individuals with a lower standing dorsal arch height and/or a wider standing midfoot width had greater mobility in their foot. In addition, those individuals with higher Foot Posture Index (FPI) values demonstrated greater mobility and those with lower FPI values demonstrated less mobility. Finally, the amount of foot mobility that an individual has can be predicted reasonably well using either a 3 or 4 variable linear regression model.

**Conclusions:**

Because of the relationship between static foot posture and mobility, it is recommended that both be assessed as part of a comprehensive evaluation of a individual with foot problems.

## Background

It is not uncommon for a person's foot posture and mobility to be assessed during a clinical examination. In such situations, the clinician uses both foot posture and foot mobility to evaluate the person's overall foot function as well as to assist them in the proper management of a variety of different foot pathologies. Unfortunately, the exact relationship between foot posture and mobility is not well established and therefore the value of assessing both features has not been validated. In addition, the most valid and useful measures to determine foot posture or mobility in a clinical examination have not been established.

A number of different methods have been described in the literature to quantify or classify standing foot posture. The Foot Posture Index (FPI) has been proposed as a fast, simple method of visually classifying foot postures as either pronated, supinated or normal based upon six different visual foot posture criteria [1]. The FPI has demonstrated moderate to good intra-rater and inter-rater reliability as well as criterion validity [2,3]. Furthermore, classification of foot posture based upon the FPI has shown an association with the development of various overuse injuries of the lower extremity and osteoarthritis of the knee [4-6]. The FPI has also been shown to have both a weak [7,8] as well as a strong [9] relationship to dynamic foot function. In addition to the FPI, the height of the dorsum of the foot measured at 50% of the person's total foot length and the ratio of dorsal foot height to foot length have also been proposed to quantify static foot posture [10]. Studies looking at the reliability of these measurements have shown that they have good intra-rater and inter-rater reliability [11]. In addition, these values were collected on a relatively large number of subjects to create normative values. Such measurements, particularly arch height, have also been associated with the development of lower extremity overuse injuries [12-14].

The dorsal arch height ratio, first proposed by Williams and McClay [15] is the ratio between the vertical height of the dorsum of the foot measured at 50% of the total length of the foot to the truncated or ball length. McPoil et al [10] demonstrated that the dorsal arch height ratio when measured in bilateral standing with equal weight placed on each foot provided the clinician with a reliable and valid method to classify static foot posture. These authors also provided normative values for the dorsal arch height ratio for 850 subjects.

Assessment of foot mobility has received less attention in the literature, but typically has been assessed either with the navicular drop or navicular drift test. Brody first described the navicular drop test in 1982. It is a measure of sagittal plane mobility of the midfoot as measured by the vertical change in the height of the navicular tuberosity [16]. Research into the clinical application of the navicular drop test has demonstrated that a relationship does exist between the magnitude of the vertical change in the navicular tuberosity and the development of various lower extremity injuries [13,17-19]. Although the navicular drop test has been shown to have good intra-rater reliability, it has either poor to moderate inter-rater reliability [20-22]. In addition, despite its relatively widespread use, it lacks normative data from a large cohort of healthy individuals. In response to these concerns with the navicular drop test, McPoil et al described an alternative method of measuring vertical change of the arch. By assessing the change in the dorsum of the arch rather than the navicular tuberosity during weight bearing and non-weight bearing, they demonstrated good to high levels of intra-rater and inter-rater reliability and were valid when compared to radiographs [10]. In addition, McPoil and colleagues noted that the greater the vertical change in dorsal arch height, the greater the amount of foot mobility and provided normative values from 345 subjects [11].

In a kinematic analysis of the navicular bone, Cornwall and McPoil demonstrated that the navicular bone not only moves in a vertical direction during the stance phase of gait, but in the medial-lateral direction as well, especially during the later portion of the stance phase [23]. The navicular drift test was first described as a way to quantify this medial-lateral movement of the midfoot [24]. Although the navicular drift test has been shown to have moderate to high intra-rater reliability ICC values, it is also accompanied by large standard errors of the measurement [25,26]. In 2009, McPoil et al described a method of assessing medial-lateral movement of the midfoot in both weight bearing and non-weight bearing that did not require palpation of the navicular tuberosity. In their study of 345 healthy individuals, they reported very high intra-rater and inter-rater reliability values for what they termed the difference in midfoot width [11]. They further noted that an increase in the difference in midfoot width, caused by greater medial-lateral midfoot motion, was indicative of increased foot mobility. In the same paper they also described a measurement called the foot mobility magnitude, which represented the composite value for both the difference in dorsal arch height (or vertical change in arch mobility) as well as the difference in midfoot width (or change in medial-lateral midfoot mobility) [11].

Although it is intuitive to assume that an individual with a high arch foot posture would have decreased foot mobility, the opposite may not be true for an individual with a low arch foot posture. The individual with a low arch foot posture could indeed exhibit increased foot mobility or have actually decreased mobility as in the case of a rigid pes planus foot deformity. Hoppenfeld [27] described what he termed a "test for rigid or supple flat feet" based on observing the foot in sitting and then in standing in an attempt to help clinicians delineate the degree of foot mobility of an individual with a low arch foot posture. While it is generally accepted that low arched or "pronated" feet are more mobile and high arched or "supinated" feet are less mobile, minimal evidence exists substantiating this relationship. Thus the purpose of this study was two-fold. The first purpose of this study is to determine the relationship between four reliable measures of static foot posture in comparison to three reliable measures of foot mobility. The second purpose is to determine which measurements of static foot posture could be considered the best predictors of the magnitude of foot mobility. We hypothesized that feet with minimal foot mobility would have a high arched static foot posture, whereas feet with low arch static foot posture would have increased foot mobility in a population of healthy subjects without foot pathology.

## Methods

### Subjects

A convenience sample of 203 healthy subjects was recruited for the current study. The demographic information for the subjects who participated in this study can be found in Table 1. None of the subjects had pain in their lower extremity or foot and ankle for at least 6 months prior to participating in the study. Subjects were excluded if they presented with an antalgic gait or physical limitation due to a lower extremity musculoskeletal injury or condition that might significantly alter either the morphology or mobility of their foot. In addition, subjects were excluded if they had a significant history of a lower extremity trauma. The Institutional Review Board at Northern Arizona University approved the study and all subjects gave their written informed consent before participating in the study.

**Table 1:** Mean demographic Information on the Subjects Recruited for this Study Values in parentheses are standard deviations.

	N	AGE (yrs)	HEIGHT (cm)	WEIGHT (kg)
**MALE**	85	26.7 (4.5)	179.2 (7.9)	81.7 (11.6)

**FEMALE**	118	24.8 (3.3)	165.7 (6.5)	64.1 (9.8)

**TOTAL**	203	25.6 (3.8)	171.3 (7.1)	71.3 (10.6

### Foot posture assessment

**Figure 1:**
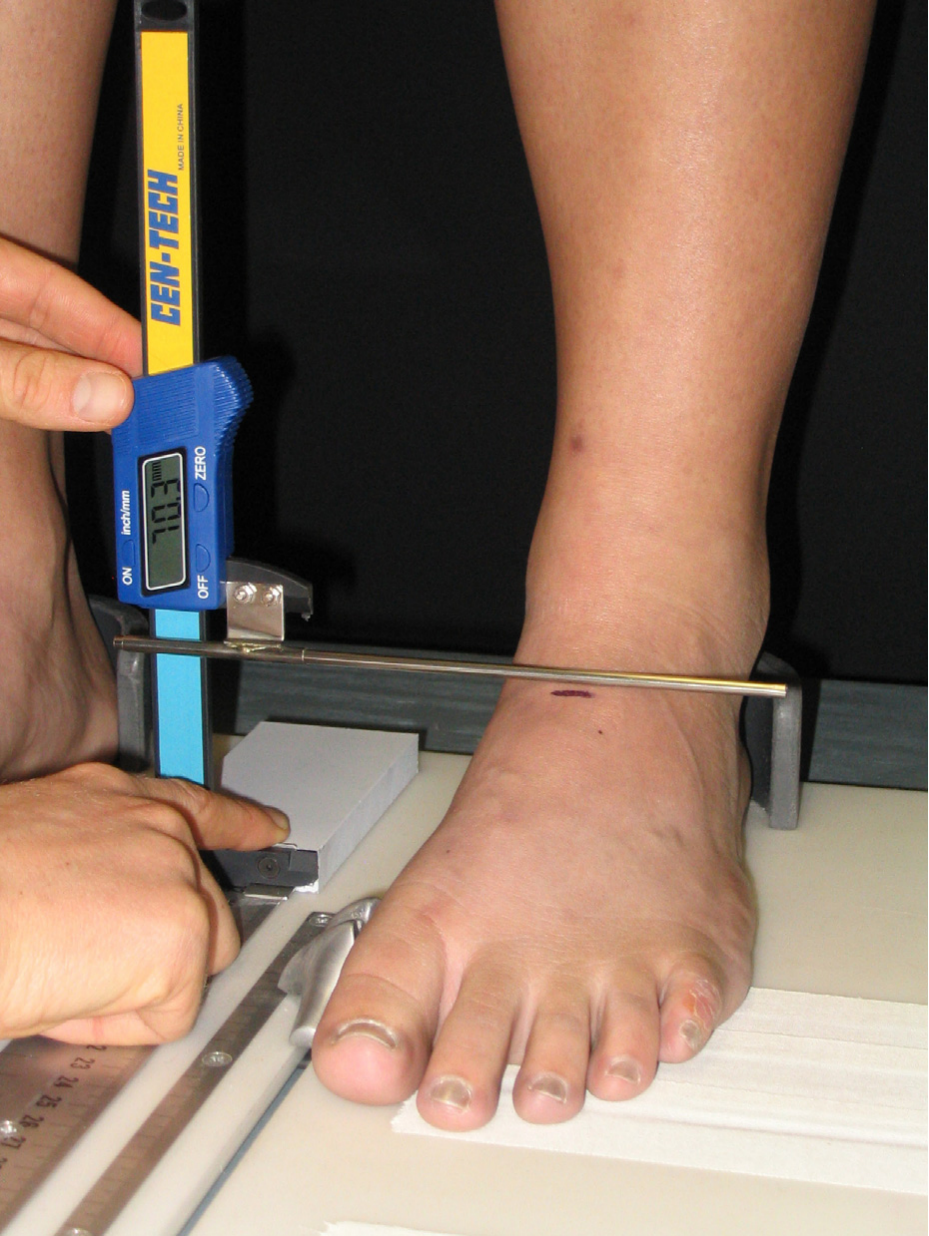
**Measurement of dorsal arch height during standing using a digital gauge**.

**Figure 2:**
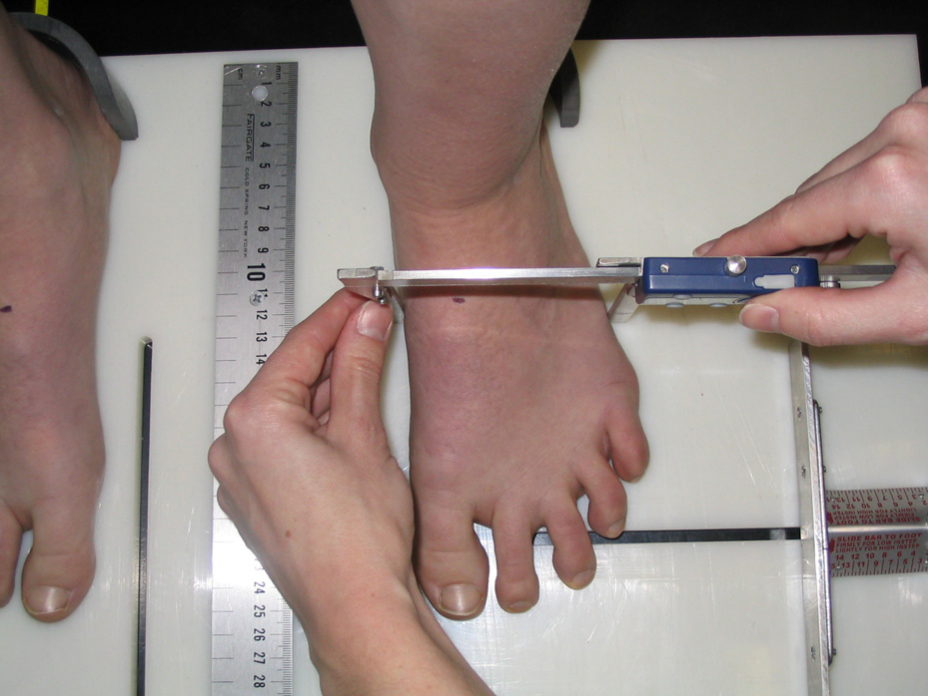
**Measurement of midfoot width during standing using a modified digital calliper**.

**Figure 3:**
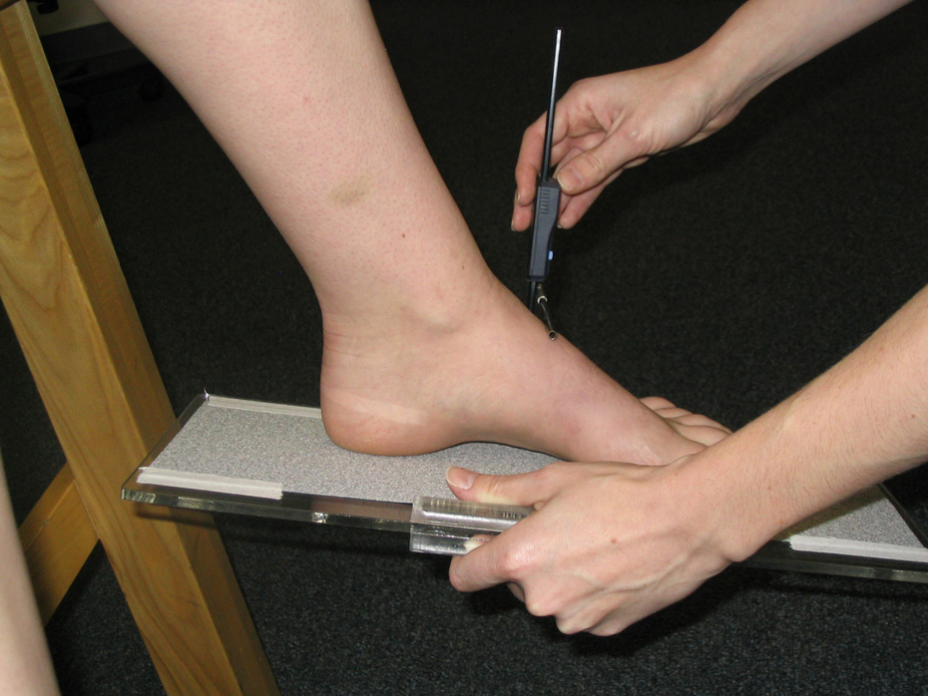
**Measurement of dorsal arch height while non-weight bearing using a digital gauge**.

**Figure 4:**
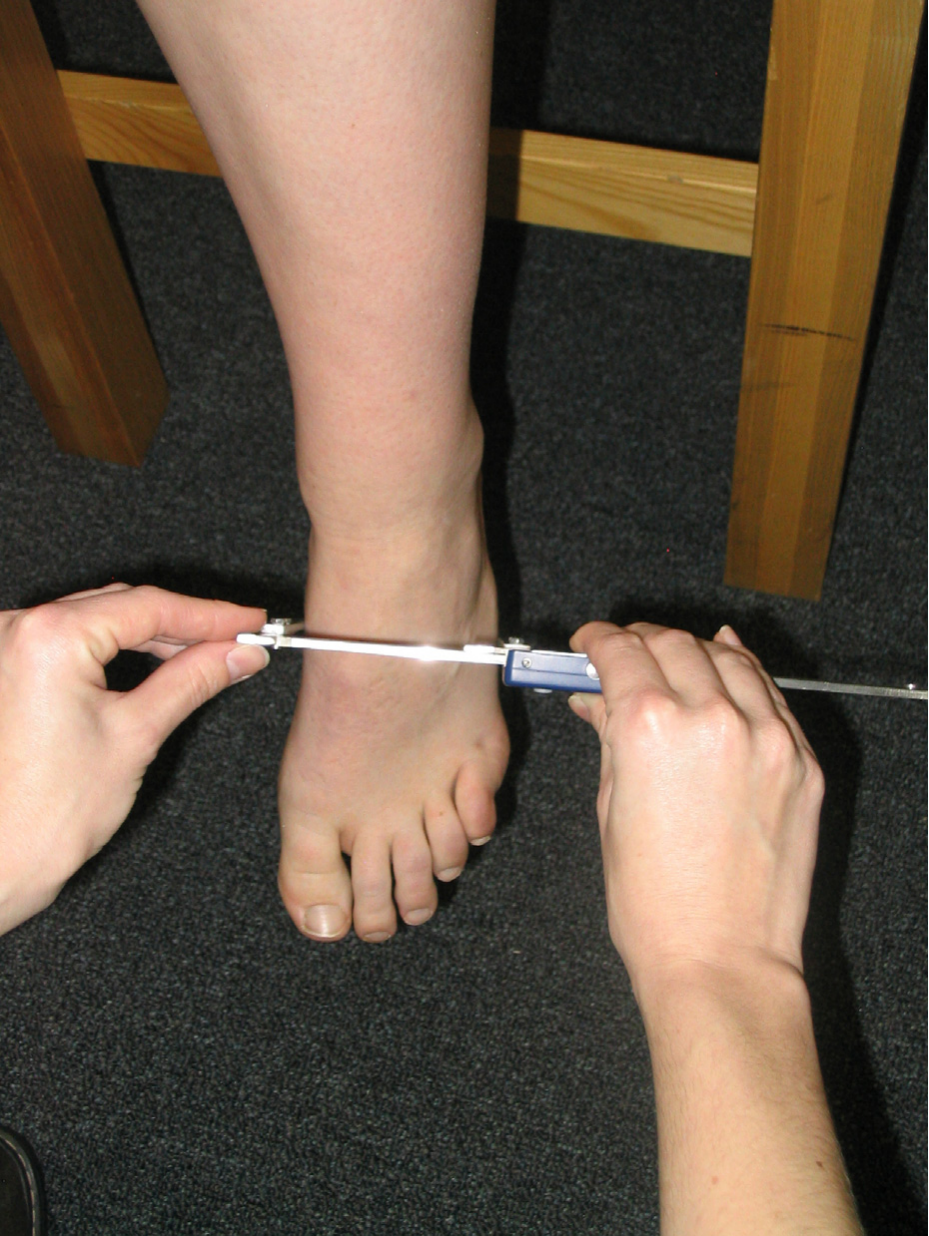
**Measurement of midfoot width while non-weight bearing using a modified digital calliper**.

The four measures of static foot posture that were used in this investigation included the Foot Posture Index, the dorsal arch height, the dorsal arch height ratio, and midfoot width. The six-variable Foot Posture Index (FPI-6) was used to characterize the static foot posture of each subject. The FPI-6 has previously been shown to have good inter-rater reliability and moderate intra-rater reliability [2]. The same procedure for scoring of the FPI-6 that has been described in the literature was followed in this study [8]. Basically, this procedure involved asking the subject to take several steps in-place, prior to settling into a comfortable stance position. While each subject stood in their relaxed stance position with their arms by their side and looking straight ahead each of the 6 clinical criteria of the FPI-6 were assessed and a scored on a 5-point scale from -2 to +2 by the same individual (TM). The six criteria were, position of the head of the talus, observation of the curves above and below the lateral malleoli, the extent of calcaneal inversion/eversion, the extent of the bulge in the region of the talonavicular joint, the congruence of the medial longitudinal arch and the extent of abduction/adduction of the forefoot on the rearfoot [8]. A negative score indicated "supination" and a positive score indicated "pronation". The 6 scores were then summed to give each subject a composite score ranging from -12 to +12. In addition to FPI-6, each subject's dorsal arch height (DAH), dorsal arch height ratio (DAHR), and midfoot width (MFW) was measured while they stood in their relaxed stance position using the protocol previously described by McPoil and associates [10]. With each subject standing with equal weight on both feet, the DAH and MFW were measured at fifty percent of the total foot length using a digital calliper (Model #93293, Cen-Tech, Harbor Freight Tools, Carmarillo, CA 93011). See Figure 1 and 2. Prior to obtaining the standing measurements, each subject was positioned so that both heels were 15.24 cm apart. The subject was then instructed to place equal weight on both feet during the measurements. Calculating the ratio of DAH to the subject's total foot length derived the DAHR variable. Once the weight bearing measurements were obtained, the subject was asked to sit on the end of a table so that both lower legs were non-weight bearing and the ankles slightly plantar-flexed. Placing a portable plastic platform with a digital calliper attached to it under, but not touching the plantar surface of the foot, the dorsal arch height in non-weight bearing was measured. Care was taken so that the portable platform did not forcibly push the subject's foot into ankle dorsiflexion. When the subject indicated that the portable platform was "just touching" the plantar surface of their foot, the vertical digital calliper attached to the portable platform was used to measure the height of the dorsal arch at 50% of the total foot length (see Figure 3). To measure the midfoot width in non-weight bearing, a digital calliper was positioned so that the edges of the two metal plates attached to each pin of the calliper where aligned laterally and medially to the 50% length point on the dorsum of the right foot and just made contact with the skin of the foot (see Figure 4). All measurements were performed by the same individual (TM) who had over three years of experience performing each of the tests performed in this study. In addition, all of these variables have previously been shown to have high intra-rater and intra-rater reliability [11].

### Foot mobility assessment

The foot mobility of each subject was assessed using three different variables; Difference in Dorsal Arch Height (DAHDIFF), Difference in Midfoot Width (MFWDIFF) and the Foot Mobility Magnitude (FMM). The dorsal arch height in weight bearing was subtracted from the dorsal arch height measured in non-weight bearing to determine the DAHDIFF. The midfoot width measured in non-weight bearing was subtracted from the midfoot width obtained in weight bearing to determine the MFWDIFF. The FMM is a composite measure of both DAHDIFF and MFWDIFF and involves taking the square root of the sum of each variable after it has been squared. All of these variables have been described previously in the literature and have been shown to have good to high intra-rater and inter-rater reliability [11]. Again, the same individual who had over three years of experience performing the tests (TM) performed all of the above measurements and the procedure described in the literature was followed.

### Data analysis

Descriptive statistics were first calculated for each of the static foot posture and mobility variables measured. After verifying that each variable was normally distributed based upon demonstrated non-significant skewness or kurtosis [28], each subject's foot was classified as having either "minimal", "normal" or "excessive" foot mobility on each of the 3 foot mobility variables using plus or minus one standard deviation from the mean value. Using DAH, each subject's foot was also classified as having either "low", "normal", or "high" arches using plus or minus one standard deviation from the mean value. In addition, each subject's MFW value was used to classify the subject's foot as being "wide", "normal", or "narrow" based upon plus or minus one standard deviation from the mean value. Finally, each subject's foot was classified as being "pronated", "normal", or "supinated" based upon plus or minus one standard deviation from the mean using the normative values reported by Redmond, Crane and Henz [29]. These normative values were used to minimize the possibility that the FPI-6 values obtained in the current study did not have the same distribution as has been reported in the literature using a much bigger sample.

To determine the relationship between foot posture and foot mobility, Pearson Correlation Coefficients were first calculated between the four static foot posture and three foot mobility variables. Because Zifchock and colleagues found that the feet of women have less stiffness compared to men [30], a series of analysis of co-variances (ANCOVA) tests were performed. In order to further investigate the possible role of the FPI-6 and its relationship to foot mobility, each person's FPI-6 value was classified as either "Severely Pronated", "Pronated", "Normal", "Supinated", or "Severely Supinated" using the criteria proposed by Redmond and associates [29]. A series of ANCOVA tests with subject gender being designated as the covariate were performed using the foot mobility measures DAHDIFF, MFWDIFF and FMM as the dependent variables. A second series of ANCOVA tests were performed on the foot mobility categorical variables using the static foot posture variables as the dependent variable. The ordinal FPI-6 data were converted to Rasch transformed scores, which allowed the data to be analyzed as interval data [31]. In addition, a third series of ANCOVA tests were performed on the foot posture categorical variables using the foot mobility variables as the dependent variable. An alpha level of .05 was used for all test of statistical significance.

Finally, a forward step-wise multiple regression analysis was performed on each of the three foot mobility variables using the four static foot posture variables. For the forward step-wise multiple regression analysis, a significance level of p < 0.05 was required for a variable to be entered into the model and p > .10 to be removed from the model. Variables were removed from the model if they were not significantly correlated (p < 0.01) with the dependent variable or if the Variance Inflation Factor (VIF) was greater than 5.0 indicating the possibility of multicollinearity. All statistical analyses were performed using PASW statistical software, version 18.0 (SPSS Inc, Chicago, IL, USA).

## Results

Table 2 contains the mean and standard deviation of each of the variables measured in this study. All of the 7 variables were determined to be normally distributed because they did not have a significant (p < 0.01) amount of either skewness or kurtosis [28]. As such, no transformation of the data was performed.

**Table 2:** Mean and Standard Deviation Values for Each of the Foot Posture and Foot Mobility Variables.

Variable	Mean	Standard Deviation
**DAH (cm)**	6.4	.57

**DAHR**	.347	.03

**MFW (cm)**	8.4	.76

**FPI-6 **(transformed)	1.8	2.4

**DAHDIFF (cm)**	1.2	.25

**MFWDIFF (cm)**	.93	.32

**FMM (cm)**	1.54	.30

The mean and standard error of each static foot posture variable for the three subgroups of each foot mobility variable is shown in Table 3. The results of the ANCOVA tests showed that with the exception of DAH within DAHDIFF classification, static foot posture was significantly different (p < 0.05) between each of the 3 subgroups of foot mobility. For example, feet having the greatest mobility as defined by either DAHDIFF, MFWDIFF, or FMM had a "lower" DAH or DAHR, a "wider" midfoot, and a more "pronated" foot posture.

**Table 3:** Mean (standard error) values for the four static foot posture variables in each of the foot mobility classifications based upon +/- one standard deviation.

	DAHDIFF			MFWDIFF			FMM		
	**Minimal (n = 59)**	**Normal (n = 295)**	**Excessive (n-52)**	**Minimal (n-72)**	**Normal (n-257)**	**Excessive (n-77)**	**Minimal (n-72)**	**Normal (n-272)**	**Excessive (n-62)**

**DAH (cm)**	6.6	6.4^a^	6.2^a^	6.7	6.4^a^	6.1^a,b^	6.6	6.4^a^	6.2^a,b^
	(.06)	(.03)	(.06)	(.06)	(.58)	(.05)	(.05)	(.03)	(.06)

**DAHR**	.362	.346^a^	.336^a,b^	.367	.347^a^	.328^a,b^	.368	.346^a^	.324^a,b^
	(.003)	(.001)	(.004)	(.003)	(.001)	(.003)	(.003)	(.001)	(.003)

**MFW (cm)**	8.1	8.4^a^	8.7^a,b^	7.7	8.4^a^	9.0^a,b^	7.8	8.4^a^	9.1^a,b^
	(.07)	(.03)	(.08)	(.06)	(.03)	(.05)	(.06)	(.03)	(.066)

**FPI-6 (transformed)**	1.3	1.8	3.0^a^	-0.3	1.7^a^	4.1^a,b^	0.3	1.8^a^	3.8^a,b^
	(.31)	(.14)	(.33)	(.27)	(.13)	(.25)	(.26)	(.13)	(.28)

^a ^indicates the value is significantly different from the Minimal value (p < .05); ^b ^indicates the value is significantly different from the Normal value (p < .05)

The mean and standard error of each foot mobility variable for the three subgroups of each foot posture variable is shown in Table 4. The results of the ANCOVA tests showed that with the exception of DAHDIFF within DAH classification, foot mobility was significantly different (p < 0.05) between each of the three categories of foot posture. For example, feet with either a "low" arch height, a "wide" midfoot, or are considered "pronated" have greater mobility as defined by DAHDIFF, MFWDIFF and FMM. The observed statistical power for test of whether there was a difference in DAHDIFF and the three classifications of DAH was 17.4%. As such, over 1000 feet would need to be included in each of the 3 classifications of DAH for the observed differences to be statistically significant. The authors therefore feel that such a small difference, even if found to be statistically significant, would not be of clinical relevance.

**Table 4:** Mean (standard error) values for the foot mobility variables in each of the static foot posture classifications based upon +/- one standard deviation.

		DAH			DAHR			MFW			FPI-6	
	**Low**	**Normal**	**High**	**Low**	**Normal**	**High**	**Narrow**	**Normal**	**Wide**	**Supinated**	**Normal**	**Pronated**

**DAHDIFF**	1.2	1.2	1.2	1.3	1.2^a^	1.1^a,b^	1.1	1.2^a^	1.3^a,b^	1.1	1.2	1.3^a,b^

	(.03)	(.01)	(.04)	(.03)	(.01)	(.03)	(.03)	(.03)	(.02)	(.03)	(.02)	(.02)

**MFWDIFF**	1.1	.9^a^	.7^a,b^	1.2	.9^a^	.7^a,b^	.6	.9^a^	1.3^a,b^	.7	.9^a^	1.1^a,b^

	(.04)	(.02)	(.04)	(.04)	(.02)	(.04)	(.03)	(.02)	(.03)	(.03)	(.02	(.02)

**FMM**	1.7	1.5^a^	1.4^a,b^	1.8	1.5^a^	1.3^a,b^	1.2	1.5^a^	1.8^a,b^	1.4	1.5^a^	1.7^a,b^

	(.04)	(.02)	(.04)	(.03)	(.02)	(.03)	(.03)	(.02)	(.03)	(.03)	(.02)	(.03)

^a ^indicates the value is significantly different from the Minimal value (p < .05); ^b ^indicates the value is significantly different from the Normal value (p < .05)

**Figure 5:**
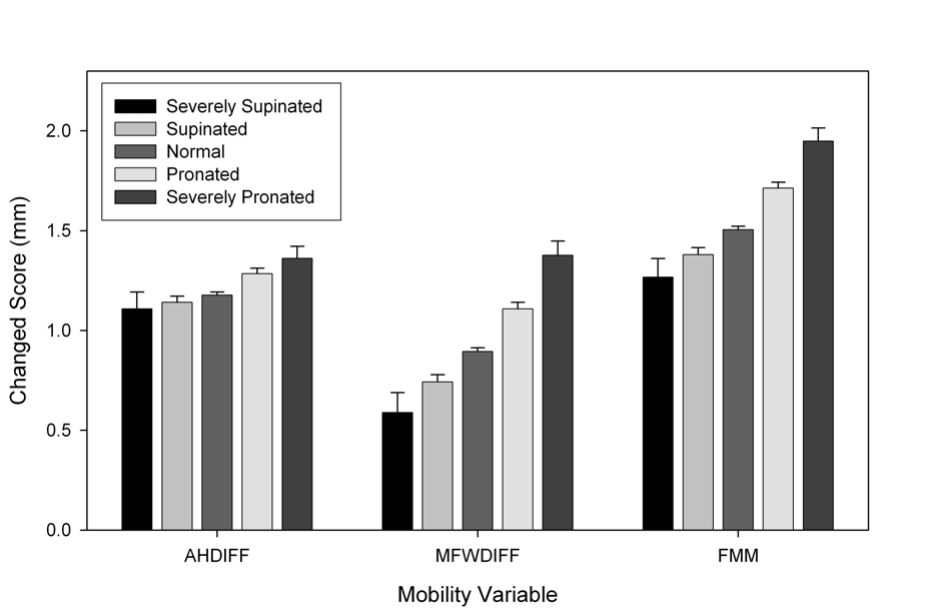
**Illustration of the relationship between FPI classification**[29]**and DAHDIFF, MFWDIFF, and FMM**.

The analysis of the more discrete categories of the FPI-6 showed that with the exception of DAHDIFF, both MFWDIFF and FMM were significantly (p < 0.05) different between for each of the five groups except for "supinated" and "severely supinated", which were not statistically different. See Table 5. The lack of statistical significance seen with the "severely supinated" group is most likely because of the small number of individuals identified as being "severely supinated" in the present sample (n = 8) and therefore had insufficient power to show a statistical difference. As can be seen in Table 5, DAHDIFF was found not to be significantly different between "severely pronated" and "pronated", between "normal" and "supinated", between "normal" and "severely supinated", and between "supinated" and "severely supinated". Figure 5 contains a plot of each of these variables across the five subcategories of the FPI-6.

**Table 5:** Mean (standard error) values for the three foot mobility variables in each of the five FPI-6 classifications from Redmond and associates.{Redmond, 2008 #1150}

	DAHDIFF	MFWDIFF	FMM
**Severely Supinated (n = 8)**	1.11 ^a, b^	.59 ^a^	1.27 ^a^
	(.08)	(.10)	(.09)

**Supinated (n = 58)**	1.14 ^b^	.74	1.38
	(.03)	(.04)	(.04)

**Normal (n = 245)**	1.17	.90	1.51
	(.02)	(.02)	(.02)

**Pronated (n = 79)**	1.29 ^c^	1.11	1.71
	(.03)	(.03)	(.03)

**Severely Pronated (n = 16)**	1.36	1.38	1.95
	(.06)	(.07)	(.07)

^a ^indicates the value is significantly different from the Supinated value (p < .05); ^b ^indicates the value is significantly different from the Normal value (p < .05); ^c ^indicates the value is significantly different from the Sever Pronated value

The result of the Pearson correlation analysis is contained in Table 6. All of the static foot posture variables were significantly (p < 0.01) correlated with each of the foot mobility variables, except for DAH to DAHDIFF and DAHG to FMM. The values for the four static foot posture measures and the three mobility measures are very similar to those reported in the literature as normative values [11,29].

**Table 6:** Pearson correlation values between the static foot posture and mobility variables measured in this study.

	DAHDIFF	MFWDIFF	FMM
**DAH**	.000	-.209 **	-.124 *

**DAHR**	-.303 **	-.462 **	-.484 **

**MFW**	.324 **	.657 **	.631 **

**FPI-6**	.193 **	.534 **	.465 **

* denotes p < 0.05; ** indicates p < 0.01

A summary of each of the 3 forward step-wise regression models is found in Table 7, 8, 9. None of the variables used in any of the models were found to cause multicollinearity and therefore were not removed from the model. Because DAH was not statistically correlated with DAHDIFF, it was not included in the model to predict that variable. As can be seen in Table 7, three variables (MFW, DAHR and DAH) were included in the regression model for DAHDIFF. The overall correlation of these three variables with DAHDIFF was moderate (R = .424) and was able to predict 18.0% of the variance of DAHDIFF. The change in the R-square value when the third variable (DAH) was added was 0.018, indicating that it could be left out of the regression model without significantly lowering the model's predictive ability. Table 8 shows the regression model for predicting MFWDIFF. As is seen, four variables (MFW, DAH, DAHR, and FPI-6) were included in the regression model for MFWDIFF. The overall correlation of these 4 variables was relatively high (R = .818) and was able to predict 67.0% of the variance of MFWDIFF. The change in the R-square value when the third and fourth variables (DAHR and FPI-6) were added was 0.010 indicating that they could be left out of the regression model without significantly lowering the model's overall predictive ability. Table 9 shows the regression model for predicting FMM. From the table in Table 9, it can be seen that the overall correlation was also relatively high (R = .740) and could predict 54.7% of the variance of FMM. The change in the R-square value when the third and fourth variables (DAHR and FPI-6) were added was 0.009 and 0.006 respectively, indicating that although statistically significant, they contributed very little to the explanation of the variance in FMM. As such, they could be left out of the regression model without significantly lowering the model's overall predictive ability.

**Table 7:** The hierarchical forward step-wise regression analysis model (F = 29.33; P < 0.001) developed to predict DAHDIFF.

Variable	Mean (SD)	Unstandardized beta (95% CI)	Standardized beta	P	R	**R**^**2**^	**R**^**2 **^**Change**	VIF
Constant	NA	1.567 (1.095, 2.387)	NA	<0.001	NA	NA	NA	NA

MFW	8.4 (0.76)	0.046 (0.005, 0.030)	0.141	0.028	.324	.105	.105	0.500

DAHR	0.35 (0.03)	-4.155 (-5.656, -10.320)	-0.441	<0.001	.402	.162	.057	0.308

DAH	6.4 (0.57)	0.107 (0.036, 0.107)	0.249	0.003	.424	.180	.018	0.285

**Table 8:** The hierarchical forward step-wise regression analysis model (F = 203.23; P < 0.001) developed to predict MFWDIFF.

Variable	Mean (SD)	Unstandardized beta (95% CI)	Standardized beta	P	R	**R**^**2**^	**R**^**2 **^**Change**	VIF
Constant	NA	-0.903 (-1.300, -2.347)	NA	<0.001	NA	NA	NA	NA

MFW	8.4 (0.76)	0.364 (0.327, 0.659)	0.863	<0.001	.657	.432	.432	2.336

DAH	.35 (.03)	-0.327 (-0.390, -0.732)	-0.583	<0.001	.806	.649	.217	3.943

DAHR	6.4 (0.57)	2.409 (1.162, 2.914)	0198	<0.001	.812	.659	.010	3.275

FPI-6 (Transformed)	1.8 (2.4)	0.018 (0.008, .028)	0.135	<0.001	.818	.670	.010	1.745

**Table 9:** The hierarchical forward step-wise regression analysis model (F = 120.86; P < 0.001) developed to predict FMM.

Variable	Mean (SD)	Unstandardized beta (95% CI)	Standardized beta	P	R	**R**^**2**^	**R**^**2 **^**Change**	VIF
Constant	NA	0.690 (0.253, 1.120)	NA	0.002	NA	NA	NA	NA

MFW	8.4 (0.76)	0.250 (0.211, 0.289)	0.632	<0.001	.631	.397	.397	2.336

DAH	.35 (.03)	-0.100 (-0.169, -0.031)	-0.191	0.004	.729	.532	.134	3.943

DAHR	6.4 (0.57)	-1.807 (-3.175, -0.439)	-0.157	0.010	.736	.541	.009	3.275

FPI-6 (Transformed)	1.8 (2.4)	0.013 (0.001, 0.025)	0.104	0.020	.740	.547	.006	1.745

## Discussion

We hypothesized that feet with minimal foot mobility would have a high arched static foot posture, whereas feet with a low arch static foot posture would have increased foot mobility in a population of healthy subjects without foot pathology. The results of our study substantiates our hypothesis by clearly demonstrating a relationship between static foot posture as measured by either dorsal arch height or midfoot width and the amount of foot mobility as measured by the change in arch height and midfoot width between non-weight bearing and weight bearing. Those subjects with greater foot mobility as measured by the FMM had lower dorsal arch heights and greater midfoot widths compared to subjects with less mobility (See Table 3). In addition, those subjects with "higher" arches or a more "narrow" midfoot had significantly less mobility as measured by the change in dorsal arch height and the change in midfoot width between weight bearing and non weight bearing (See Table 4). The non-significant finding for DAH between "normal" and "minimal" change in dorsal arch height (See Table 3) and between the three categories of dorsal arch height for DAHDIFF (See Table 4) is most likely because individual foot length was not taken into consideration. When dorsal arch height is standardized to the individual's foot length (DAHR), a statistically significant difference is found for both situations. See Tables 3, 4. This finding underscores the importance of always standardizing dorsal arch height to the overall length of the person's foot when using it during a clinical examination or research. A "wider" or more "narrow" midfoot being associated with more or less foot mobility was expected considering they are features generally associated with either pronation or supination.

Figure 5 contains a plot of each of the foot mobility variables across the five subcategories of FPI-6. As can be seen, those subjects with a more "pronated" foot posture had greater vertical, medial-lateral as well as global foot mobility compared to subjects with a more "supinated" foot posture. Such a finding would support the notion that both posture and mobility measures should be performed during a clinical examination of individuals with foot related injuries or disorders so that a more complete picture of the possible causes or factors related to their condition might be determined. The finding of a significant difference between foot mobility measurements across the five subcategories of the FPI-6 supports the use of the FPI-6 as a useful clinical tool because it not only conveys information about the person's foot posture, but their mobility as well. The lack of statistical significance for several of the FPI-6 categories with respect to DAHDIFF is consistent with what was observed in the preceding analysis (See Table 3). As such, this would indicate that vertical mobility of the foot plays a smaller role than that of medial lateral mobility.

The result of the forward step-wise regression analysis further demonstrates the relationship between static posture and mobility. The variables selected by the step-wise regression analysis to predict foot mobility included measures of arch height and midfoot width or the FPI-6. In all of the regression analyses, medial-lateral mobility of the midfoot as measured by MFWDIFF was shown to be the best indicator of such a relationship and underscores the importance of including medial-lateral midfoot movement and posture measurements as part of a comprehensive clinical examination of the foot, especially with movement related disorders. In all of the prediction models, FPI-6 was either not included in the resulting regression equation or its addition resulted in a small, but statistically significant change in the R-square value. This finding would indicate that the FPI-6 is a factor in predicting foot mobility, but not the most important one. See Tables 4, 5, 6. The low predictive ability of the FPI-6 may be related to the fact that FPI-6 is a composite of six different aspects of foot posture rather than just one such as midfoot width or dorsal arch height. Despite the finding that FPI-6 does not seem to help to explain a large percentage of the variance in foot mobility measures, it has the advantage of providing an overall characterization of foot posture; it is easily measured and requires no specialized equipment to perform. As such, use of the FPI-6 provides valuable clinical information and should not be excluded from a comprehensive physical examination based upon the results of the current study. The ability to predict FMM has the advantage that it is a composite measure of both vertical and medial-lateral foot mobility rather than just one. As such, it provides a more global view of total foot mobility.

A limitation of the current study involves the fact that only young health individuals were included in the study. Although restriction of the subject pool allowed the normal relationship between foot posture and mobility to be documented, it is unclear how such a relationship may be altered because of either injury or disease. The measures of foot posture and mobility used in the current study, however, can be used to study such relationships and further research in this area is warranted. In addition, although there is a clear relationship between foot posture and mobility, clinicians should not assume that everyone with a particular foot posture has the same amount of mobility.

## Conclusions

The relationship between static foot posture and foot mobility was investigated in 203 healthy individuals. Based upon the results of this study, individuals with increased vertical or medial-lateral mobility tend to have lower dorsal arch heights and greater midfoot widths compared to those with less foot mobility. In addition, foot mobility may be predicted with reasonable accuracy using a combination of midfoot width, dorsal arch height and overall foot posture classification using the FPI-6. It is recommended that the measurements used in this study to assess both foot posture and mobility should be assessed during a clinical examination to provide the clinician with a more complete understanding of the patient's foot and the possible nature of their problem.

## Competing interests

The authors have applied for a patent on the device used to quantify foot posture and mobility.

## Authors' contributions

MWC and TGM conceived of the study, and participated in its design and coordination and helped to draft the manuscript. TGM carried out the data collection. MWC performed the statistical analysis. All authors read and approved the final manuscript.

## References

[B1] The Foot Posture Index: User guide and manual http://www.leeds.ac.uk/medicine/FASTER/

[B2] Cornwall MW McPoil TG Lebec M Vicenzino B Wilson J Reliability of the Modified Foot Posture Index J Am Podiatr Med Assoc 2008 98 7 13 18202328 10.7547/0980007

[B3] Redmond A Burns J Crosbie J Ouvrier R An initial appraisal of the validity of a criterion based, observational clinical rating system for foot posture J Orthop Sports Phys Ther 2001 31 160

[B4] Burns J Keenan AM Redmond A Foot type and overuse injury in triathletes J Am Podiatr Med Assoc 2005 95 235 241 15901809 10.7547/0950235

[B5] Cain LE Nicholson LL Adams RD Burns J Foot morphology and foot/ankle injury in indoor football J Sci Med Sport 2007 10 311 319 10.1016/j.jsams.2006.07.012 16949867

[B6] Reilly K Barker K Shamley D Newman M Oskrochi GR Sandall S The role of foot and ankle assessment of patients with lower limb osteoarthritis Physiotherapy 2009 95 164 169 10.1016/j.physio.2009.04.003 19635335

[B7] Nielsen RG Rathleff M Kersting UG Simonsen O Moelgaard C Jensen K Olesen GG Lundbye-Christensen S Kaalund S The predictive value of the foot posture index on dynamic function J Foot Ankle Res 2008 1 Suppl 1 O37 10.1186/1757-1146-1-S1-O37

[B8] Redmond AC Crosbie J Pack RJ Development and validation of a novel rating system for scoring foot posture: Foot Posture Index Clin Biomech 2006 21 89 98 10.1016/j.clinbiomech.2005.08.002 16182419

[B9] Chuter VH Relationships between foot type and dynamic rearfoot plane motion J Foot Ankle Res 2010 3 9 10.1186/1757-1146-3-9 20550714 PMC2894016

[B10] McPoil TG Cornwall MW Vicenzino B Teyhen DS Molloy JM Christie DS Collins N Effect of using truncated versus total foot lenght to calculate the arch height ratio Foot 2008 18 220 227 10.1016/j.foot.2008.06.002 20307441

[B11] McPoil TG Vicenzino B Cornwall MW Collins N Warren M Reliability and normative values for the foot mobility magnitude: a composite measure of vertical and medial-lateral mobility of the midfoot J Foot Ankle Res 2009 2 1 12 10.1186/1757-1146-2-1 19267907 PMC2656480

[B12] Cowan DN Jones BH Robinson JR Foot morphologic characteristics and risk of exercise-related injury Arch Fam Med 1993 2 773 777 10.1001/archfami.2.7.773 7906597

[B13] Reilly KA Barker KL Shamley D Sandall S Influence of Foot Characteristics on the Site of Lower Limb Osteoarthritis Foot Ankle Int 2006 27 206 211 16539904 10.1177/107110070602700310

[B14] Williams DS III McClay I Hamill J Arch structure and injury patterns in runners Clin Biomech 2001 16 341 347 10.1016/S0268-0033(01)00005-5 11358622

[B15] Williams DS McClay IS Measurements used to characterize the foot and the medial longitudinal arch: reliability and validity Phys Ther 2000 80 864 871 10960934

[B16] Brody DM Techniques in the evaluation and treatment of the injured runner Orthop Clin North Am 1982 13 541 558 6124922

[B17] Bandholm T Boysen L Haugaard S Zebis MK Bencke J Foot medial longitudinal-arch deformation during quiet standing and gait in subjects with medial tibial stress syndrome J Foot Ankle Surg 2008 47 89 95 10.1053/j.jfas.2007.10.015 18312915

[B18] Bennett JE Reinking MF Pluemer B Pentel A Seaton M Killian C Factors contributing to the development of medical tibial stress syndrome in high school runners J Orthop Sports Phys Ther 2001 31 504 510 11570734 10.2519/jospt.2001.31.9.504

[B19] Loudon JK Jenkins W Loudon KL The relationship between static posture and ACL injury in female athletes J Orthop Sports Phys Ther 1996 24 91 97 8832472 10.2519/jospt.1996.24.2.91

[B20] Picciano AM Rowlands MS Worrell T Reliability of open and closed kinetic chain subtalar joint neutral positions and navicular drop test J Orthop Sports Phys Ther 1993 18 553 558 8220414 10.2519/jospt.1993.18.4.553

[B21] Evans AM Copper AW Scharfbillig RW Scutter SD Williams MT Reliability of the foot posture index and traditional measures of foot posture J Am Podiatr Med Assoc 2003 93 203 213 12756311 10.7547/87507315-93-3-203

[B22] Schultz S Nguyen D-M Windley T Kulas AS Botic T Beynnon B Intratester and intertester reliabiity of clinical measures of lower extremity anatomic characteristics: Implications for muticenter studies Clin J Sports Med 2006 16 155 161 10.1097/00042752-200603000-00012 16603886

[B23] Cornwall MW McPoil TG Relative movement of the navicular bone during normal walking Foot Ankle Int 1999 20 507 512 10473062 10.1177/107110079902000808

[B24] Menz HB Alternative techniques for the clinical assessment of foot pronation J Am Podiatr Med Assoc 1998 88 119 129 9542353 10.7547/87507315-88-3-119

[B25] Billis E Katsakiori E Kapodisrias C Kapreli E Assessment of foot posture: Correlation between different clinical techniques Foot 2007 17 65 72 10.1016/j.foot.2006.09.005

[B26] Vinicombe A Raspovic A Menz HB Reliability of navicular displacement measurement as a clinical indicator of foot posture J Am Podiatr Med Assoc 2001 91 262 268 11359892 10.7547/87507315-91-5-262

[B27] Hoppenfled S Physical Examination of the Spine and Extremities 1976 1 New York: Appleton-Century Crofts

[B28] Tabachnick BG Fidell LS Using Multivariate Statistics 1983 New York: Harper & Row, Publishers

[B29] Redmond AC Crane YZ Menz HB Normative values for the Foot Posture Index J Foot Ankle Res 2008 1 1 9 10.1186/1757-1146-1-6 18822155 PMC2553778

[B30] Zifchock RA Davis I Hillstrom H Song J The effect of gender, age, and lateral dominance on arch height and arch stiffness Foot Ankle Int 2006 27 367 372 16701058 10.1177/107110070602700509

[B31] Keenan AM Redmond AC Horton M Conaghan PG Tennant A The foot posture index: rasch analysis of a novel, foot-specific outcome measure Arch Phys Med Rehabil 2007 88 88 93 10.1016/j.apmr.2006.10.005 17207681

